# Patterns of hybridization and cryptic introgression among one- and four-needled pinyon pines

**DOI:** 10.1093/aob/mcaa045

**Published:** 2020-03-28

**Authors:** Ryan Buck, Sandra Hyasat, Alice Hossfeld, Lluvia Flores-Rentería

**Affiliations:** Department of Biology, San Diego State University, San Diego, CA, USA

**Keywords:** Cryptic introgression, genomics, hybridization, needle number, pines, pinyon resin canals

## Abstract

**Background and Aims:**

Pinyon pine hybridization is widely acknowledged, but the frequency of and contributors to such interspecific mating remain largely unstudied. *Pinus quadrifolia* has three to four needles per fascicle, suggesting that it is a result of hybridization between the five-needled *P. juarezensis* and the single-needled *P. monophylla.* In this study we address the taxonomic validity of *P. juarezensis*, the hybrid origin of *P. quadrifolia* and the presence of hybridization and intermediate morphology as a result of interspecific hybridization in this complex.

**Methods:**

We address these questions by combining a genomic and morphological approach. We generated 1868 single nucleotide polymorphisms (SNPs) to detect genetic clusters using principal co-ordinates analyis, discriminant analysis of principal components, fastSTRUCTURE and ADMIXTURE analyses, and performed a morphological analysis of the leaves.

**Key Results:**

We found that the five-needled pinyons did not differ genetically from the four-needled *P. quadrifolia*, reducing the status of *P. juarezensis* to *P. quadrifolia*. We also found no evidence that *P. quadrifolia* is of hybrid origin from *P. juarezensis* × *P. monophylla* but is instead a genetically distinct species with natural needle number variation that has yet to be explained. Hybridization does occur in this complex, but mostly between *P. quadrifolia* and *P. californiarum*, and less commonly between *P. quadrifolia* and *P. monophylla*. Interestingly, some hybrid derivatives were detected between both single-needled taxa, *P. monophylla* and *P. californiarum*, a hybrid combination that has not yet been proposed. Hybrids have intermediate morphology when they have similar genetic contributions from both parental species; however, when one parent contributes more, hybrid derivatives resemble the parent with higher genetic contribution, resulting in cryptic introgression.

**Conclusions:**

Our detailed sampling across the distribution of this complex allows us to describe the patterns of hybridization among these taxa, resolves an ancient taxonomic conflict and provides insights into the challenges of exclusively using morphological traits when identifying these taxa with cryptic hybridization and variable morphology.

## INTRODUCTION

Interspecies hybridization is relatively common across plant taxa and is thought to be the cause of several major speciation events (Soltis and Soltis, 2009; Abbott *et al.*, 2013). Hybridization tends to occur when species lack strong reproductive isolating mechanisms (Bigelow, 1965), which normally act as barriers to reproduction and help in the process of speciation (Rieseberg and Willis, 2007). If species have loose genetic barriers (compatible zygotes) and at least partially overlap spatially and temporally, then hybrids are likely to form (Zhao *et al.*, 2014). Intermediate forms can be detected in recent hybridization events (Zavarin *et al*., 1980; Delaporte *et al.*, 2001); however, when backcrossing occurs in a hybrid system, morphological traits are present either as a continuum between the two parental species (Holman *et al.*, 2003), or more representative of one of the parents, resulting in cryptic introgression (Pfenninger *et al.*, 2002; Jasińska *et al.*, 2010; Neri *et al*., 2018). The use of morphological traits to determine hybridization events can be helpful when divergent features exist between parental species, but, when morphological limits are not well defined in taxonomically challenging groups, it becomes difficult to distinguish intermediacy from interpopulation variation (Wei *et al.*, 2015). For example, in some systems, hybridization leads to a mosaic of forms or even to extreme or novel characters (Rieseberg *et al.*, 1993).

**Fig. 1.**
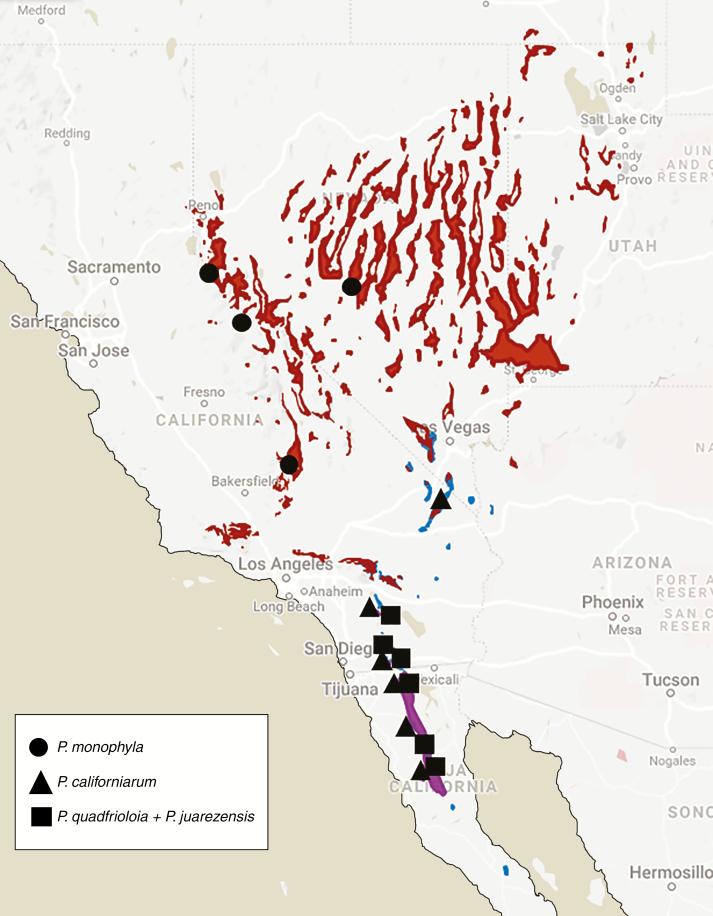
Putative distribution map of *P. monophylla* (red), *P. californiarum* (blue) and *P. quadrifolia + P. juarezensis* (purple) (USGS, 1999; Cole *et al.*, 2008). Sampling locations are represented by the overlaid shapes (circle = *P. monophylla*, triangle = *P. californiarum*, square = *P. quadrifolia + P. juarezensis*).

Hybridization in pines has been widely acknowledged (Critchfield, 1975, 1986; Willyard *et al.*, 2009; Menon *et al*., 2018, 2020). The pinyon pine complex is an excellent system to study hybridization due to its lack of: strong reproductive isolating mechanisms (Lanner, 1974*a*); taxonomic congruence (Gernandt *et al*., 2001, 2003); and conclusive genetic studies (Montes *et al.*, 2019). Most pine species lack interspecific incompatibility mechanisms (Critchfield, 1975) and have wind-mediated, long-distance pollen dispersals (Williams, 2010) that can facilitate gene flow between allopatric groups (Wright, 1952). Most notably, the Parry pinyon pine, *Pinus quadrifolia* Parl. ex Sudworth, has been proposed to have a hybrid origin based on morphological features and geography (Lanner, 1974*a*). *Pinus quadrifolia* commonly has four needles per fascicle, as its epithet suggests, and occurs from Riverside County, California, USA to northern Baja California, Mexico. Based on intermediate morphology, Lanner (1974*a*) hypothesized its origin as the result of interspecific hybridization between two species with similar pollen dispersal times (Malusa, 1992; Farjon and Styles, 1997) and partially overlapping distributions (Fig. 1), *P. monophylla* Torrey & Fremont and *P. juarezensis* Lanner. At the time of his discovery, needle number was the main morphological trait used in classifying pinyon pines, so he reasoned that individuals with one needle per fascicle were one species (*P. monophylla*), individuals with five needles were another (*P. juarezensis*) and the three- and four-needled individuals (*P. quadrifolia*) were intermediate hybrids of the former two.

Several attempts have been made to resolve the hybrid nature *of P. quadrifolia*; however, they have provided inconclusive results. The idea of hybridization was explored using additional morphological features other than needle number, but these studies focused on the potential hybridization between *P. monophylla* and *P. quadrifolia* without considering *P. juarezensis* (Zavarin *et al.*, 1980; Snajberk *et al.*, 1982). These studies also explored chemical profiles, such as mono- and sesquiterpenoids, that could be used for species identification between *P. quadrifolia* and *P. monophylla* (Zavarin *et al.*, 1980); however, an intermediate chemical profile was detected in individuals in sympatry, suggesting hybridization. Additionally, the only genetic study including the three putative taxa (*P. juarezensis*, *P. quadrifolia* and *P. monophylla*) was done with internal transcribed spacer (ITS) sequences (Gernandt *et al.*, 2001), which have been proven to have paralogy in pines and other plants (Grimm and Denk, 2008; Xiao *et al.*, 2010; Flores-Rentería *et al*., 2013, 2017). When using this nuclear marker, species were not monophyletic, so the authors then suggested a potential hybridization event between *P. monophylla* and *P. juarezensis* or incomplete lineage sorting as the cause. Therefore, the question of whether *P. quadrifolia* has a hybrid origin has remained unanswered for >40 years.

Since Lanner’s publication suggesting the hybrid origin of *P. quadrifolia* from the parental species *P. monophylla* and *P. juarezensis* (Lanner, 1974*a*), several taxonomic studies have been conducted, some of them suggesting the splitting of *P. monophylla* into three different taxa based on morphology and niche variability: *P. monophylla*, *P. californiarum* and *P. fallax* (Little, 1968; Bailey, 1987; Cole *et al.*, 2008). One of these suggested taxa, *P. californiarum* D.K. Bailey, has more resin canals and occupies drier habitats in higher elevations than *P. monophylla*. In a recent study, hybridization between *P. californiarum* and *P. quadrifolia* was explored using a Hyb-seq method, yet no gene flow was detected (Montes *et al.*, 2019). One reason could be the lack of adequate sample size due to the taxonomic focus of the study which aimed to capture as many species as possible using only a few individuals. Montes *et al.* (2019) stated themselves that increased sampling would be required to draw accurate conclusions about admixture events in this system. In our study, we will be testing whether *P. juarezensis* hybridizes with *P. monophylla* or with the recently proposed taxa *P. californiarum.* In contrast, *P. juarezensis* has not been recognized as an independent species by some authors, but rather as a natural morphological variation of *P. quadrifolia*, able to produce 3–5 needles per fascicle. For example, Farjon and Styles (1997) challenged Lanner’s conclusion, pointing to the extreme variation in needle number on a single tree and the year to year variation on trees reported by Lanner (1974*a*) himself. We will be testing the taxonomic validity of *P. juarezensis* as an independent species.

With advancements in next-generation sequencing (NGS), the ability to detect reticulated evolution through hybridization has drastically improved (Pritchard *et al.*, 2000; Twyford and Ennos, 2012; Melville *et al.*, 2017). We are now able to revisit these hybridization studies with a finer scale, allowing us to examine the purported hybrid origin of *P. quadrifolia* and address the taxonomic standing of *P. juarezensis*. The goals of this study are: (1) to determine the taxonomic validity of *P. juarezensis* as a species; (2) to assess the hybrid origin of *P. quadrifolia*; and (3) to test for hybridization and intermediate morphology among species in this complex. We have included samples from the taxa of interest using nuclear genomic data as well as morphological data to assess these questions.

## MATERIALS AND METHODS

### Sampling

Samples were taken from 13 locations across the Southwestern USA and Baja California, Mexico (Table 1; Fig. 1), randomly sampling ten trees per putative species per site, at least 30 m apart from each other. The distribution of this complex extends from southern Idaho, USA to southern Baja California, Mexico, with a majority of *P. monophylla* occurring in western Utah, Nevada and central California, *P. californiarum* occurring in southern Nevada, southern California and down into Baja California, and *P. juarezensis* (five needle) and *P. quadrifolia* (3–4 needles) occurring in southern California and Baja California. These taxa are characterized by inhabiting small disjunct areas in southern California and Baja California. Ten centimetres of branch tips were cut from each tree, representing 2–3 years of growth and averaging approx. 50 fascicles per tree. Most collections were made from late 2017 to early 2019, with the exception of a few collections made previously (2011–2013). We collected a larger number of individuals in San Jacinto because Lanner (1974*a*) proposed this area as one of the primary hybrid zones between *P. juarezensis* and *P. monophylla.*

**Table 1.** Sample locations with population co-ordinates and number of trees sampled per population

Location	Latitude	Longitude	No. of trees genotyped	Needle type
Markleeville, CA	38.69894	–119.77082	10	*P. monophylla*
Manhattan, NV	38.54101	–117.05981	6	*P. monophylla*
Mono Lake, CA	37.92145	–119.06433	7	*P. monophylla*
Pine Mt, CA	36.02982	–118.15251	9	*P. monophylla*
Mojave Desert, CA	35.17397	–115.40707	13	*P. californiarum* Hybrids
San Jacinto, CA	33.55887	–116.60994	37	*P. quadrifolia* *P. californiarum* *P. juarezensis* Hybrids
Laguna Mts, CA	32.87554	–116.41017	9	*P. quadrifolia, P. juarezensis*
Jacumba, CA	32.63317	–116.09395	15	*P. quadrifolia* *P. californiarum* *P. juarezensis* Hybrids
La Rumorosa, Baja CA	32.521544	–116.04122	21	*P. quadrifolia* *P. californiarum* *P. juarezensis* Hybrids
San Salvador, Baja CA	31.742415	–115.97815	8	*P. quadrifolia* *P. juarezensis*
Lazaro Cardenas, Baja CA	31.257595	–115.59940	11	*P. californiarum*
Northern San Pedro Martir, Baja CA	31.034611	–115.46477	18	*P. quadrifolia* *P. californiarum* *P. juarezensis*
Southern San Pedro Martir, Baja CA	31.02131	–115.51488	10	*P. quadrifolia* *P. juarezensis*

California (CA), Nevada (NV), Baja California (Baja CA).

### Genomic clustering analyses

In order to examine the taxonomic validity of *P. juarezensis* as a species, the hybrid origin of *P. quadrifolia* and hybridization among species of this complex, we first identified genetic clustering and admixed individuals. Nuclear DNA was extracted using the 2 % cetyltrimethylammonium bromide (CTAB) protocol (Doyle and Doyle, 1987), quantified and sent to Diversity Arrays Technology (DArT), who produced a reduced library, and sequenced on an Illumina Hiseq2500 system (DArTseq). DArTseq™ represents a combination of DArT complexity reduction methods and NGS platforms (Jaccoud *et al.*, 2001). Genome reduction is achieved by a combination of endonucleases that specifically target low-copy DNA areas, rather than repetitive DNA fragments (Wenzl *et al.*, 2004). This allows for detection of a high number of informative single nucleotide polymorphisms (SNPs) across the genome. The result is a genomic ‘representation’, comprising both constant and polymorphic fragments across individuals. NGS of these ‘representations’ reveals the sequence (approx. 70 bp) of an informative DNA fragment and each individual’s state compared with all others, namely (1) homozygosity with the reference allele; (2) homozygosity with an alternative allele; or (3) heterozygosity, comprising both a reference and an alternative SNP allele. The technology was optimized for our taxa using combinations of enzymes (*Pst*I/*Hpa*II, *Pst*I/*Sph*I, *Sbf*I/*Hpa*II and *Sbf*I/*Mse*I) to select the most appropriate complexity reduction method, in terms of both the size of the representation and the fraction of a genome selected for assays. DArTseq™ has been successfully applied in genomic studies exploring species boundaries and hybridization in plants and animals (Von Mark *et al.*, 2013; Melville *et al.*, 2017, Rutherford *et al.*, 2018). This technique enables genome-wide studies of non-model organisms, those for which there is limited genomic information. For our analysis, we selected *P. lambertiana* as the reference genome (GCA_001447015.2) due to its relatively close evolutionary relationship to the pinyon subsection *Cembroides*. A low-density DArTseq assay resulted in 18 518 SNPs. These SNPs have an index generated by reproducing the data independently, which is ‘the proportion of technical replicate assay pairs for which the marker score is consistent’ (Gruber *et al.*, 2019). We filtered out all loci with reproducibility lower than 100 %, missing data lower than 15 %, all monomorphs, all loci departing from Hardy–Weinberg equilibrium and all but one locus where there was more than one locus per sequence tag, resulting in a final data set of 1868 loci. Input data were the metadata provided by DArTseq, saved as an xlsx file: ‘0’ (homozygosity with reference allele); ‘1’ (homozygosity with alternative allele); ‘2’ (heterozygote) and ‘–’, fragment missing in representation – double null (absence of fragment with an SNP in genomic representation). The processed marker data were reformatted into appropriate file types for downstream analyses using the R program dartR (Gruber *et al.*, 2018).

The 1868 loci were included in four complementary methods of genetic clustering: principal co-ordinates analysis (PCoA), discriminant analysis of principal components (DAPC), fastSTRUCTURE and ADMIXTURE, which also allowed us to detect admixed individuals between different genetic clusters. We then evaluated whether *P. juarezensis* (five-needled individuals) forms its own genetic cluster and whether *P. quadrifolia* (three- and four-needled individuals) is formed by admixed individuals from the parental species *P. monophylla* and *P. juarezensis* or whether there are other pairs of species hybridizing in this complex.

#### PCoA

A PCoA, which considers differences in allele frequencies between individuals, was performed in dartR (gl.pcoa.plot) to examine genetic distance among populations (Gruber *et al.*, 2019).

#### DAPC

Population clustering was determined using a DAPC (Jombart *et al.*, 2010) in R with the adegenet package (Jombart, 2008). DAPC uses a model-free k-means clustering algorithm on a principal components analysis (PCA)-transformed data set, which maximizes variation between groups while reducing the number of variables and computation time needed to identify existing genetic clusters (Jombart and Collins, 2015). Differing cluster solutions are compared using the Bayesian information criterion (BIC), with the lowest BIC score corresponding to the optimal cluster solution. The xvalDapc command, which uses a stratified random sampling to ensure one member of each population is represented in both a training set consisting of 90 % of the data and a validation set consisting of the remaining 10 % of the data, was used as a cross-validation method to determine the appropriate number of principal components to retain (Jombart and Collins, 2015).

#### fastSTRUCTURE.

A Bayesian analysis of population clustering was performed in the software fastSTRUCTURE (Raj *et al.*, 2014) using the logistic prior and five cross-validations. Output files include the mean Q value for each individual, defining the mean probability as belonging to any one of the populations K1 to Kx. Model complexity (*K*) was selected using the chooseK command built into fastSTRUCTURE. The resulting Q mean bar plots were visualized using the online application pophelper (Francis, 2017). Results were confirmed using ADMIXTURE (Alexander *et al.*, 2009) and a sub-structuring method, wherein samples with the same population identity (*K*) were reanalysed in fastSTRUCTURE using the logistic prior and five cross-validations to observe lower hierarchical structuring (Raj *et al.*, 2014).

### Hybrid generation identification

#### NewHybrids.

In order to determine an individual’s hybrid category, e.g. early F_1_ or advanced generation (F_2_ and backcross), we used a Bayesian model-based clustering method in the software NewHybrids 1.0 (Anderson and Thompson, 2002). The program uses Markov chain Monte Carlo simulations to compute the posterior probability of an individual belonging to pre-defined ancestry categories, including pure, F_1_, F_2_ or backcrossed (Table 2). The program compares two parental genotypes at a time, so we created three data sets to represent each pairwise species cross by removing individuals with ancestry of the third genotype (e.g. for *P. monophylla* × *P. quadrifolia* analysis, we removed pure *P. californiarum* individuals and individuals with *P. californiarum* ancestry). Runs were initiated at different random starting points using the Jeffrey’s prior for both theta and pi with a burn-in of 10 000 and 100 000 sweeps (Couch *et al.*, 2016).

**Table 2.** Twelve genotype frequency categories input into NewHybrid analyses based on Chhatre *et al.*, (2018)

Genotype category	Cross type	Expected ancestry proportions			
		AA	Aa	aA	aa
Pure	Species 1	1	0	0	0
Pure	Species 2	0	0	0	1
F_1_	Species 1 × Species 2	0	0.5	0.5	0
F_2_	F_1_ × F_1_	0.25	0.25	0.25	0.25
F_1_ Backcross 1	F_1_ × Species 1	0.5	0.25	0.25	0
F_1_ Backcross 2	F_1_ × Species 2	0	0.25	0.25	0.5
F_2_ Backcross 1	F_2_ × Species 1	0.5	0.125	0.125	0.25
F_2_ Backcross 2	F_2_ × Species 2	0.25	0.125	0.125	0.5
1 Backcross × F_1_ Backcross 1	Species 1 × (F_1_ × Species 1)	0.75	0.125	0.125	0
2 Backcross × F_1_ Backcross 2	Species 2 × (F_1_ × Species 2)	0	0.125	0.125	0.75
1 Backcross × F_2_ Backcross 1	Species 1 × (F_2_ × Species 1)	0.625	0.125	0.125	0.125
2 Backcross × F_2_ Backcross 2	Species 2 × (F_2_ × Species 2)	0.125	0.125	0.125	0.625

### Morphology

In order to determine which morphological features are consistent with the genetic clusters and to detect intermediate morphology associated with hybridization, we analysed a range of leaf morphological traits that have been used in most taxonomic studies of pinyon pines, including needle number, number of resin canals and number of rows of stomata (Little, 1968; Bailey, 1987; Lanner and Phillips, 1992; Malusa, 1992; Christensen *et al.*, 1995; Cole *et al.*, 2008; Flores-Rentería *et al.*, 2013). All sample shoots were kept in the freezer at –20 °C and analysed individually at room temperature. All fascicles found in the 10 cm tip branches were visually examined for needle number. Most trees retain their needles, but some drop a few needles. To avoid any bias in needle number estimates, close examination of branch tips was done under a stereomicroscope to inspect fascicle scars, allowing us to detect dropped needles and accurately estimate the range of needle number per branch. Then we identified whether trees have the same number of needles across the branch (uniform number) or if they have a range of needle numbers (non-uniform). One fascicle was measured from branch tips with uniform needles per fascicle numbers. For branch tips with non-uniform needles per fascicle numbers, one fascicle of each needle number variant was measured. Clear nail polish was used when necessary to enhance the visualization of stomatal rows. A stomatal position index was created ranging from 1 to 5, where 1 means ventral position only, 2 is full ventral and partial dorsal, 3 is complete on both sides, 4 is full dorsal and partial ventral, and 5 is dorsal only. In addition to stomatal position, cross-sectional area and thickness of hypodermal layers were determined (Flores-Rentería *et al.*, 2013). Every needle sample was cross-sectioned a few times with a razor blade in the midsection and rehydrated with water before measuring the cross-sectional area, dermal thickness and number of dermal layers. Measurements for cross-sectional area were traced in micrometres, and thickness of hypodermal layers was recorded as the average of four measurements around the needle. Variation in the number of resin canals in needles from different fascicles of the same branch was initially observed, thus two resin canal measurements were taken; however, the difference between these two measurements proved to be non-significant so only the first measurement was included in further analyses. All measurements were done using an ultra-high-resolution Nikon SMZ25 stereoscopic microscope zoom ×0.5–1.6 and NIS Elements software. We used Microsoft Excel to plot the means and standard errors of needle number by resin canals and stomatal rows by resin canals per population with and without genetic hybrids, following Cole *et al.* (2008). This method allows us to examine differences among species while showing the intraspecific variation present among populations. It also allows us to examine the morphology of hybrids and compare them with general species characteristics.

## RESULTS

### Genetic clustering analyses

**Fig. 2.**
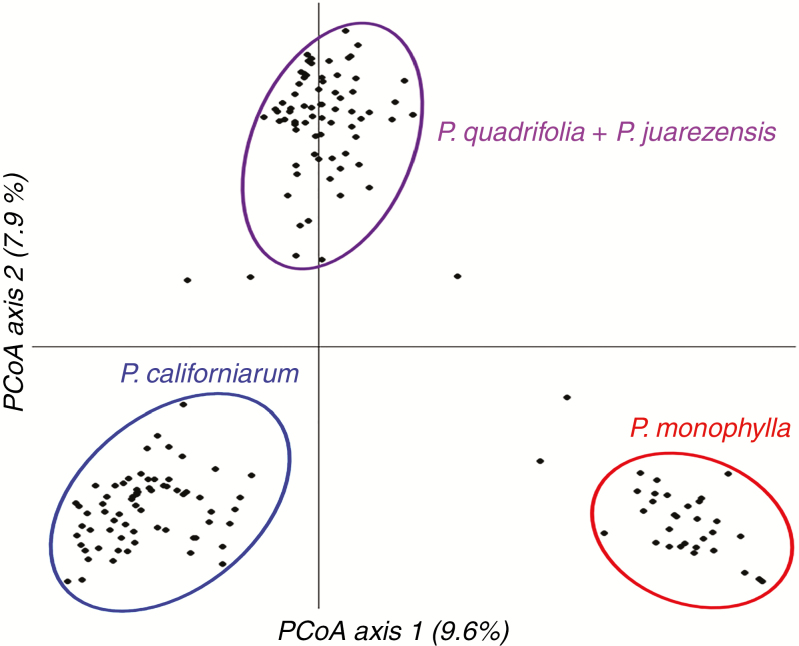
Principal co-ordinates analysis showing the variation in genetic distance with three main groups circled by genetic identity. Each black dot represents an individual; dots outside of circles represent potential hybrids and hybrid derivatives.

The PCoA (Fig. 2) shows individuals separating into three groups, with PCoA axis one representing 9.6 % of variation in genetic distance and PCoA axis two representing 7.9 %. While those two axes represent the two most informative axes, the subsequent axes incrementally represent less variation, with PCoA axis three representing 1.5 %, four representing 1.3 %, and so on. Each group represents individuals that have the morphology of *P. monophylla*, *P. quadrifolia*/*P. juarezensis* and *P. californiarum*, respectively. Individuals with allele frequencies in between *P. monophylla* and *P. quadrifolia*, *P. monophylla* and *P. californiarum*, and *P. quadrifolia* and *P. californiarum* can be seen falling out of the three main groups, suggesting possible admixture among the three taxa. It is important to note that a PCoA does not present statistical clusters, but instead functions as a tool to visualize differences in allele frequency and therefore must be interpreted with caution. Individuals on the PCoA graph may group together due to similarities in allele frequencies, but a PCoA does not provide a group assessment as in K-means clustering methods duch as DAPC and fastSTRUCTURE.

**Fig. 3.**
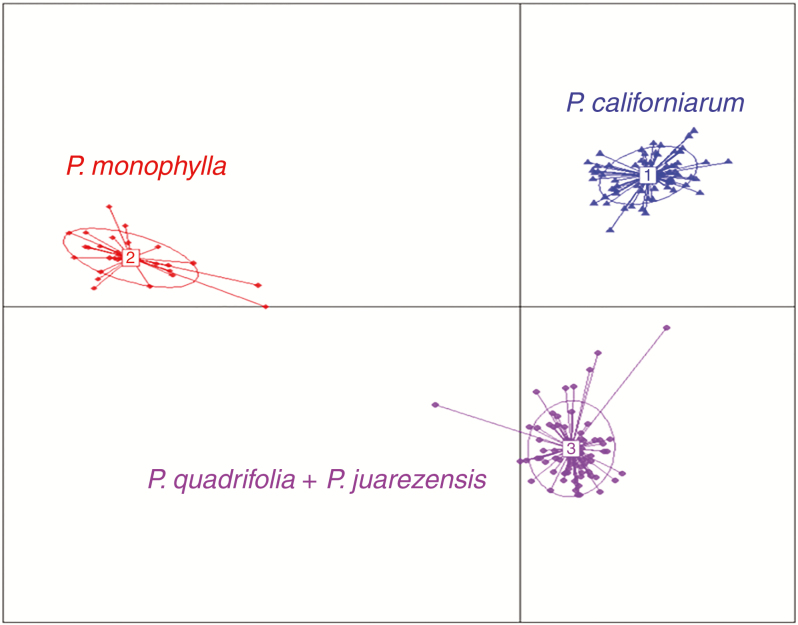
Discriminant analysis of principal components showing three distinct genetic clusters coloured by genetic identity. Each dot represents an individual connected to the genetic cluster’s centroid by a line; inertia ellipses indicate their assignment to one of the three genetic clusters inferred by DAPC.

The DAPC analysis (Fig. 3) of the SNP data set resulted in a value of *K* = 3 by K-means clustering, retaining 20 principal components and two discriminant functions. All sample populations of *P. monophylla* clustered together as one group (coloured in red). All sample populations of *P. quadrifolia* and purported *P. juarezensis* clustered together as one group (coloured in purple). All sample populations of *P. californiarum* (coloured in blue) clustered together as one group.

**Fig. 4.**
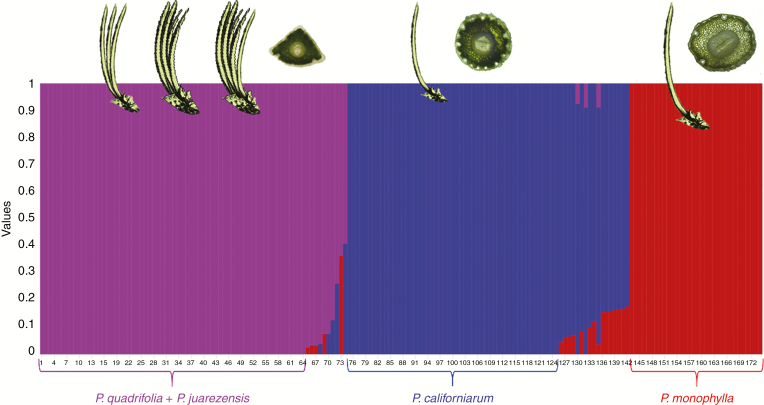
fastSTRUCTURE plot showing three genetic clusters (*K* = 3) coloured by genetic identity. Each line on the *x*-axis represents an individual, and the probability of those individuals belonging to a certain genetic cluster is represented by the proportion of colour on the *y*-axis. Lines with multiple colours represent individuals with admixture from multiple genetic clusters. Cartoons showing the number of needles per fascicle and cross-sectional pictures showing the resin canals are overlaid on their corresponding genetic groups.

Our analyses of fastSTRUCTURE show three distinct genetic clusters using the chooseK method and visualized in pophelper (Fig. 4; Supplementary data Fig. S1), supporting the DAPC and PCoA results. Again, *P. monophylla* forms one cluster (coloured in red), *P. quadrifolia* and purported *P. juarezensis* another cluster (coloured in purple) and *P. californiarum* the third cluster (coloured in blue). These results show no genetic distinction between individuals with morphological characteristics of *P. quadrifolia* (three to four needles) and *P. juarezensis* (five needle); this was further confirmed by the sub-structuring analysis of the cluster containing only the putative *P. juarezensis* and *P. quadrifolia* (Supplementary data Fig. S2). Moreover, this analysis did not support the hypothesized hybrid origin of *P. quadrifolia*. However, these results do show extensive admixture among the three taxa, with several examples of *P. monophylla* × *P. quadrifolia* (as in San Jacinto), *P. monophylla* × *P. californiarum* (as in Mojave) and *P. quadrifolia* × *P. californiarum* (as in Rumorosa, Mojave, San Jacinto and Jacumba) admixture events. These admixture events are evident in the fastSTRUCTURE plot (Fig. 4) where individuals have more than one genetic cluster assigned to them. ADMIXTURE analyses supported three genetic clusters with the lowest cross-validation error at *K* = 3 (Supplementary Data Fig. S3). Sub-structuring using fastSTRUCTURE’s logistic prior and ten cross-validations found no lower hierarchical structuring below *K* = 3.

### Hybrid generation identification

Interestingly, the NewHybrids analyses (Table 3) show that most individuals initially identified as having hybrid ancestry in fastSTRUCTURE end up grouping as advanced generation backcrosses, suggesting that F_1_ hybrids are able to reproduce further. Several individuals had multiple category assignments with probability scores lower than 0.98, suggesting uncertainty in assignment (Anderson and Thompson, 2002). These pure and mixed assignment individuals are possibly backcrosses of a more advanced generation than our genotype categories can detect. Individuals that appeared near a Q value of 0.4 on the fastSTRUCTURE plot were at least third- or fourth-generation hybrid derivatives, with the most recent hybrid ancestry generation of F_2_ × *P. quadrifolia* and no F_1_ or F_2_ individuals detected. A majority of the individuals were identified as fourth-generation backcrosses, having a genotype class of Backcross × (F_2_ × Backcross).

**Table 3.** Levels of ancestry determined by NewHybrids for the apparent hybrid derivatives indicated in fastSTRUCTURE analyses.

NewHybrids assignment	Contributing species	No. of individuals
Pure *P. quadrifolia*	*P. quadrifolia*	61
Pure *P. monophylla*	*P. monophylla*	32
Pure *P. californiarum*	*P. californiarum*	53
*P. quadrifolia* × (F_2_ × *P. quadrifolia*)	*P. monophylla*, *P. quadrifolia*	7
*P. quadrifolia* × (F_2_ × *P. quadrifolia*)	*P. californiarum*, *P. quadrifolia*	4
F_2_ × *P. Quadrifolia*	*P. monophylla*, *P. quadrifolia*	1
F_2_ × *P. Quadrifolia*	*P. californiarum*, *P. quadrifolia*	1
*P. californiarum* x (F_2_ × *P. californiarum*)	*P. monophylla*, *P. californiarum*	14

### Morphological analysis

**Fig. 5.**
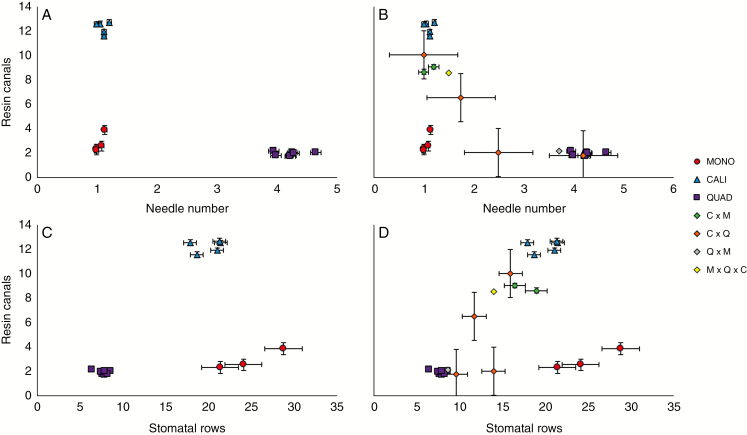
Morphological analyses showing in (A and B) needle number by resin canals and (C and D) stomatal rows by resin canals, with (A and C) showing pure species, and (B and D) showing pure species and hybrid derivatives. Each dot represents the mean of a population with standard error bars; each colour corresponds to a species (red circle = *P. monophylla*, blue triangle = *P. californiarum*, purple square = *P. quadrifolia*, diamonds = hybrid derivatives).

In order to properly assign morphological traits to species, characteristics were assigned to the genetic groups resulting from the genetic structuring analyses, particularly fastSTRUCTURE and NewHybrids. Individuals assigned exclusively to one genetic cluster were determined to be pure, while individuals of mixed assignment were run through NewHybrids and distinguished as hybrids (F_1_) and hybrid derivatives (F_2_, backcrosses or advanced generation backcrosses). All morphological results are listed in Table 4 and distinguishing traits are plotted in Fig. 5. Morphological results also supported three groups, with *P. monophylla* and *P. californiarum* having one needle per fascicle on average but separated by the larger number of resin canals in *P. californiarum.* In contrast, *P. quadrifolia + P. juarezensis* formed one group, having the highest number of needles but the lowest number of rows of stomata, and stomata just on the ventral surface. Intermediate forms were detected when hybrids and hybrid derivatives were plotted (Fig. 5B, C); however, in some cases, some individuals more closely resembled one of the parental species.

**Table 4.** Morphological characteristics based on genetic identity

Genetic identity	Needle per fascicle		Resin canals		Stomatal rows	Stomatal position	Area (mm^2^)	No. of dermal layers	Thickness of dermal layers (µm)
	Mean	Range	Mean	Range					
*P. monophylla*	1.06 ± 0.24	1–2	2.82 ± 1.08	1–6	24.48 ± 4.09	Complete	1.9 × 10^3^ ± 7.0 × 10^2^	2.36 ± 0.65	59.08 ± 13.86
*P. quadrifolia*	4.19 ± 0.77	2–5	1.91 ± 0.78	0–7	7.69 ± 1.80	Ventral only	7.9 × 10^2^ ± 2.5 × 10^3^	2.59 ± 0.55	36.76 ± 37.57
*P. californiarum*	1.11 ± 0.31	1–2	12.30 ± 2.96	6–23	19.81 ± 4.02	Complete	1.1 × 10^3^ ± 2.1 × 10^3^	2.49 ± 0.50	37.43 ± 25.25
M × Q	3.71 ± 1.03	2–5	2.04 ± 0.26	2–3	8.71 ± 1.98	Ventral only or complete	5.6 × 10^2^ ± 3.6 × 10^2^	2.14 ± 0.64	40.37 ± 13.90
Q × C	2.83 ± 1.40	1–5	4.22 ± 4.60	1–17	11.58 ± 3.90	Ventral only or complete	5.9 × 10^2^ ± 4.0 × 10^2^	2.66 ± 0.47	38.49 ± 18.42
C × M	1.13 ± 0.33	1–2	8.94 ± 2.65	1–12	16.92 ± 2.56	Complete	2.0 × 10^3^ ± 2.4 × 10^3^	2.97 ± 0.62	51.38 ± 12.86
M × Q × C	1.50 ± 0.50	1–2	8.50 ± 0.50	8–9	14.00 ± 0.00	Complete	8.8 × 10^2^ ± 9.5 × 10^1^	2.00 ± 0.00	51.38 ± 0.84

Note that *P. juarezensis* is treated as a synonym of *P. quadrifolia* because no genetic differences were found between them.

Letters in the hybrid cross rows represent the first initial of the species name (M = *P. monophylla*, Q = *P. quadrifolia*, C = *P. californiarum*).

## DISCUSSION

Genetic clustering analyses show three groups: *P. monophylla*, *P. californiarum* and *P. quadrifolia*. Five-needled individuals are not genetically distinguishable from three- and four-needled individuals, contrary to Lanner’s original hypothesis. Individuals with hybrid ancestry were detected; however, the hypothesized hybrid origin of *P. quadrifolia* from the species tested was not supported. Morphological analyses showed a clear distinction between *P. californiarum* and *P. monophylla* in the number of resin canals, while both *P. californiarum* and *P. monophylla* were distinguishable from *P. quadrifolia* in the number of needles per fascicle. *Pinus quadrifolia* showed variation in the number of needles per fascicle throughout its range, with most individuals having a variable number of needles on the same branch.

Lanner’s hypotheses on the existence of *P. juarezensis* and the hybrid origin *P. quadrifolia* proved to be incorrect. While *P. quadrifolia* does have variation in its needle number, this variation is not explained by interspecific hybridization. *Pinus juarezensis* is not a valid taxon, but rather a synonym of *P. quadrifolia*, as five-needled individuals genetically cluster with the three- and four-needled individuals. Hybridization was detected between *P. quadrifolia* and *P. monophylla*, as well as between *P. quadrifolia* and *P. californiarum*. Interestingly, a new admixture combination was detected between *P. californiarum* and *P. monophylla*. The morphological traits of recent hybrid derivatives seemed to be intermediate between the parental species, yet, as apparent backcrossing occurred, the advanced generation hybrid derivatives appeared morphologically indistinguishable from their main genetically contributing parent.

### Genetic clustering analyses do not support *P. juarezensis* as a valid taxon

Previous studies have either failed to include *P. juarezensis* in their analyses or found inconclusive results regarding its taxonomic validity (Zavarin *et al.*, 1980; Snajberk *et al.*, 1982; Gernandt *et al.*, 2001; Montes *et al.*, 2019). In our study, we included several populations with the five-needled pinyon individuals, including some from Lanner’s original discovery (Lanner, 1974*a*). Genetic analyses, including PCoA, DAPC, fastSTRUCTURE and ADMIXTURE, showed three distinct genetic groups, with no difference between *P. quadrifolia* and the purported *P. juarezensis*. This may initially appear to align with Lanner’s hypothesis that the five-needled taxon would eventually be introgressed out of existence (Lanner, 1974*a*); however, purely five-needled trees were sampled and did not have a distinct genetic structure from the three- and four-needled samples identified as *P. quadrifolia*, implying that they are all one species. With pine trees’ long generation time and slow mutation rate (Buschiazzo *et al.*, 2012; De La Torre *et al.*, 2017), it is not plausible that a species could have disappeared from the gene pool since Lanner’s observation (Lanner, 1974*a*) as a result of genomic swamping. It is more likely that *P. quadrifolia* can express 3–5 needles per fascicle. Additionally, purely five-needled trees were so rare compared with the mixed three-, four- and five-needled individuals that they seem to be more of an exception, rather than the standard for a species. Lanner himself reported such rarity in his initial study, with only three individuals having 100 % five needle coverage for 1 year but then expressing three or four needles in subsequent years (Lanner, 1974*a*). With all this evidence considered, we reduce *P. juarezensis* to a synonym of *P. quadrifolia*, supporting the hypothesis of Farjon and Styles (1997).

### *Pinus quadrifolia* is not of *P. monophylla* × *P. juarezensis* hybrid origin

The unique needle number variation (i.e. 3–5 needles per fascicle) present in *P. quadrifolia* populations has led many to question its origin. Hybridization events between other pinyon species, such as *P. monophylla* and *P. edulis* for example, produce individuals with mixed needle number (Lanner, 1974*b*; Lanner and Phillips, 1992; Cole *et al.*, 2008). Previous research explored the possibility that the same mechanisms could be producing such variation in *P. quadrifolia*. For the first time, our study used NGS methods to test for the hybrid origin of *P. quadrifolia* suggested by the intermediate morphology between five-needled and one-needled pinyon pines. Genetic analyses showed no evidence that *P. quadrifolia* was of *P. monophylla* × *P. juarezensis* hybrid origin, but instead has a unique genetic identity. FastSTRUCTURE results showed *P. quadrifolia* as a distinct genetic group, with most individuals lacking genetic admixture. Individuals with a combination of three, four and five needles were most common, yet showed no genetic distinction from the individuals with only four or only five needles. We therefore reject Lanner’s declassification of the Parry pinyon to the hybrid epithet *P. × quadrifolia* and support its species status as *P. quadrifolia*. The morphological variation originally seen by Lanner and others is probably not due to hybridization events but may be explained by intraspecies variation or environmentally induced plasticity. In fact, in several tree stands where *P. quadrifolia* and *P. californiarum* grow together, the needle variation is not increased compared with isolated pure *P. quadrifolia* stands but instead this needle number range occurs throughout its distribution. This variation could be environmentally related, yet *P. californiarum* shares a large portion of its distribution with *P. quadrifolia* and does not seem to have nearly as much variation; however, the mode of monophylly inheritance is unknown (Gabilo and Mogensen, 1973).

### Tridirectional hybridization: detection of hybridization among the valid taxa *P. quadrifolia*, *P. californiarum* and *P. monophylla*

Our data support the existence of three taxa: *P. quadrifolia*, *P. californiarum* and *P. monophylla.*Lanner (1974*a*) treated the one-needled individuals from Southern California and Baja California (San Jacinto Mountains, Rumorosa and San Pedro Martir) as *P. monophylla*. We found that these populations are actually *P. californiarum* based on the genetic data. Furthermore, the morphology of these populations matches the morphology of other *P. californiarum* populations, with a high number of resin canals. We found no evidence of *P. monophylla* in Baja California. While *P. quadrifolia* is not of *P. monophylla* × *P. juarezensis* hybrid origin, it hybridizes with *P. californiarum* and *P. monophylla*. Previous studies hypothesized that hybridization occurred between *P. quadrifolia* and *P. monophylla* (Zavarin *et al.*, 1980; Snajberk *et al.*, 1982; Kral, 1993); however, we also discovered genetic admixture between *P. quadrifolia* and *P. californiarum*. These two species share a significant amount of their range and even have stands where they grow a few metres apart. It appears that there are no strong genetic/post-zygotic barriers preventing hybrids of these species from forming and proliferating within natural populations. However, more extensive pre- and post-zygotic studies examining shifted phenology and outbreeding depression are needed to understand why some populations have more hybrid derivatives than others. A few instances of *P. quadrifolia* hybridizing with *P. monophylla* were seen in San Jacinto, and, while these species’ ranges do not overlap geographically, their nearest distributions are only 30 miles apart, a feasible distance for both pollen and seeds dispersed by corvids to travel (Wells, 1983; Williams, 2010). This southern direction of gene flow would correspond to the Santa Ana winds that occur through October to May (Muhs *et al.*, 2007), well within the pollen dispersal times of *P. monophylla* and *P. quadrifolia* around May (Malusa, 1992; Farjon and Styles, 1997). Given the lower frequency of *P. monophylla* genes present in *P. quadrifolia*, these individuals may be relics from the early Holocene when *P. monophylla* had a more restricted southernly range, a pattern of distribution that has been detected in some populations of *P. monophylla* and its close relative *Pinus edulis* (Madsen and Rhode, 1990; Duran *et al.*, 2012). This contraction in distribution has been considered the source of hybridization in the latter (Duran *et al.*, 2012).

Surprisingly, a majority of the introgression detected in this study was between two species not previously thought to hybridize, *P. monophylla* and *P. californiarum*. Originally considered synonymous, the potential of these two species for hybridization has long been overlooked, possibly due to their identical needle number. They share a significant portion of their range in southern Nevada and southern California, but differ quite drastically in their number of resin canals. Curiously, in the site where hybrid derivatives are present (Mojave National Park), only one *P. californiarum* individual was detected, indicating how readily these two species hybridize. This could additionally imply heterosis, with increased fertility of the F_1_ generation allowing for frequent backcrossing, or selection potentially favouring hybrid phenotypes, promoting the proliferation of individuals with hybrid ancestry. Future studies on the fitness of *P. monophylla* × *P. californiarum* crosses must be done to explore these hypotheses.

### Morphological traits in pure species vs. hybrids

Our study delimited species by genetic clusters first, without any *a priori* bias towards morphological traits. We then generalized the average characteristics of each genetic group to assign morphological qualities to each species. In doing so, our analyses were not influenced by strict morphological cut-offs and we were able to more accurately identify individuals with hybrid ancestry and intraspecific variation compared with other studies in pinyon pines. It is important to note that while some distinguishing morphological patterns were found, these should be used as suggestions for species identification, rather than strict species guidelines/boundaries.

Interestingly, the morphology of all three hybrid types largely depends on the species contributing more of its genotype. For example, in *P. quadrifolia × P. californiarum* hybrid derivatives, individuals with a majority of *P. californiarum* genotype tend to have one to two needles per fascicle with over ten resin canals; whereas individuals with a majority of *P. quadrifolia* genotype tend to have two to four needles per fascicle and two to four resin canals. This implies that needle morphology is largely genetically controlled and may be less influenced by environment than previously thought. The only exceptions to this were in recent hybrid derivatives, whose traits were more intermediate between their parents. For example, one *P. monophylla* × *P. quadrifolia* hybrid derivative found in San Jacinto had exclusively two needles per fascicle, 12 stomatal rows and two resin canals, resembling *P. edulis*. It is possible that as these individuals of hybrid ancestry further backcross, the intermediate traits could be recombined or introgressed out to more closely represent one of the parental species. This unfortunately makes the identification of individuals with advanced generation hybrid ancestry quite difficult without using genetic methods because these hybrid derivatives are often indistinguishable from their main genetically contributing parent, concordant with other hybrid systems (Rieseberg *et al.*, 1993).

This study provides an insight into the morphological and genetic outcomes of species undergoing gene flow. We plan to further explore these outcomes and patterns of hybridization in pinyon pines, especially in our newly discovered admixture combination of *P. monophylla* and *P. californiarum*. While our study addresses long-standing taxonomic issues within a small group of pinyon pines, the NGS methods and genetics first approach we used can be applied to a variety of taxa with controversial standings and cryptic introgression.

## SUPPLEMENTARY DATA

Supplementary data are available online at https://academic.oup.com/aob and consist of the following. Figure S1: fastSTRUCTURE bar plots of a growing number of genetic clusters, with the software-determined best K framed. Figure S2: the sub-structuring of *P. quadrifolia* and *P. juarezensis* samples in fastSTRUCTURE, showing one genetic cluster. Figure S3: the ADMIXTURE analysis of all samples, showing three genetic clusters corresponding to *P. monophylla*, *P. californiarum* and *P. quadrifolia* + *P. juarezensis*.

mcaa045_suppl_Supplementary_Figure_1

mcaa045_suppl_Supplementary_Figure_2

mcaa045_suppl_Supplementary_Figure_3

## FUNDING

This work was supported by the Hispanic-Serving Institutions Education Grants (HSI) Program [grant no. 2018-38422-28614/project accession no. 1016839] from the USDA National Institute of Food and Agriculture, by the National Science Foundation [grant no. DEB-0816675] and by the University Grants Program of San Diego State University.

## References

[CIT0001] Abbott R, Albach D, Ansell S, et al 2013 Hybridization and speciation. Journal of Evolutionary Biology 26: 229–246. 23323997 10.1111/j.1420-9101.2012.02599.x

[CIT0002] Alexander DH, Novembre J, Lange K 2009 Fast model-based estimation of ancestry in unrelated individuals. Genome Research 19: 1655–1664. 19648217 10.1101/gr.094052.109 PMC2752134

[CIT0003] Anderson EC, Thompson EA 2002 A model-based method for identifying species hybrids using multilocus genetic data. Genetics 160: 1217–1229. 11901135 10.1093/genetics/160.3.1217 PMC1462008

[CIT0004] Bailey DK 1987 A study of *Pinus* subsection *Cembroides*. I: the single-needle pinyons of the Californias and the Great Basin. Notes from the Royal Botanic Garden, Edinburgh 44: 275–310.

[CIT0005] Bigelow RS 1965 Hybrid zones and reproductive isolation. Evolution 19: 449–458.

[CIT0006] Buschiazzo E, Ritland C, Bohlmann J, Ritland K 2012 Slow but not low: genomic comparisons reveal slower evolutionary rate and higher dN/dS in conifers compared to angiosperms. BMC Evolutionary Biology 12: 8. doi: 10.1186/1471-2148-12-8. 22264329 10.1186/1471-2148-12-8 PMC3328258

[CIT0007] Chhatre VE, Evans LM, DiFazio SP, Keller SR 2018 Adaptive introgression and maintenance of a trispecies hybrid complex in range-edge populations of *Populus*. Molecular Ecology 27: 4820–4838. 30071141 10.1111/mec.14820

[CIT0008] Christensen KM, Whitham TG, Keim P 1995 Herbivory and tree mortality across a pinyon pine hybrid zone. Oecologia 101: 29–36. 28306972 10.1007/BF00328896

[CIT0009] Cole KL, Fisher J, Arundel ST, Cannella J, Swift S 2008 Geographical and climatic limits of needle types of one- and two-needled pinyon pines. Journal of Biogeography 35: 257–269. 21188300 10.1111/j.1365-2699.2007.01786.x PMC3001037

[CIT0010] Couch AJ, Unmack PJ, Dyer FJ, Lintermans M 2016 Who’s your mama? Riverine hybridisation of threatened freshwater Trout Cod and Murray Cod. PeerJ 4: e2593. 27812407 10.7717/peerj.2593 PMC5088581

[CIT0011] Critchfield WB 1975 Interspecific hybridization in *Pinus*: a summary review. In: Fowler DP, Yeatman CY eds. Symposium on Interspecific and Interprovenance Hybridization in Forest Trees.Proceedings of the 14th Meeting of the Canadian Tree Improvement Association, Part II, 99–105.

[CIT0012] Critchfield WB 1986 Hybridization and classification of the white pines (*Pinus* section *Strobus*). Taxon 35: 647–656.

[CIT0013] Delaporte KL, Conran JG, Sedgley M 2001 Interspecific hybridization within *Eucalyptus* (Myrtaceae): subgenus *Symphyomyrtus*, sections *Bisectae* and *Adnataria*. International Journal of Plant Sciences 162: 1317–1326.

[CIT0014] De La Torre AR, Li Z, Van de Peer Y, Ingvarsson PK 2017 Contrasting rates of molecular evolution and patterns of selection among gymnosperms and flowering plants. Molecular Biology and Evolution 34: 1363–1377. 28333233 10.1093/molbev/msx069 PMC5435085

[CIT0015] Doyle JJ, Doyle JL 1987 A rapid DNA isolation procedure for small quantities of fresh leaf tissue. Focus 12: 13–15.

[CIT0016] Duran KL, Pardo A, Mitton JB 2012 From middens to molecules: phylogeography of the piñon pine, *Pinus edulis*. Journal of Biogeography 39: 1536–1544.

[CIT0017] Farjon A, Styles BT 1997 Flora Neotropica. Pinus (Pinaceae). New York: Organization for Flora Neotropica, New York Botanical Garden.

[CIT0018] Flores-Rentería L, Rymer PD, Riegler M 2017 Unpacking boxes: integration of molecular, morphological and ecological approaches reveals extensive patterns of reticulate evolution in box eucalypts. Molecular Phylogenetics and Evolution 108: 70–87. 28185948 10.1016/j.ympev.2017.01.019

[CIT0019] Flores-Rentería L, Wegier A, Ortega Del Vecchyo D, et al 2013 Genetic, morphological, geographical and ecological approaches reveal phylogenetic relationships in complex groups, an example of recently diverged pinyon pine species (Subsection *Cembroides*). Molecular Phylogenetics and Evolution 69: 940–949. 23831459 10.1016/j.ympev.2013.06.010

[CIT0020] Francis RM 2017 pophelper: an R package and web app to analyse and visualize population structure. Molecular Ecology Resources 17: 27–32. 26850166 10.1111/1755-0998.12509

[CIT0021] Gabilo EM, Mogensen HL 1973 Foliar initiation and the fate of the dwarf-shoot apex in *Pinus monophyla*. American Journal of Botany 60: 671–677.

[CIT0022] Gernandt DS, Liston A, Piñero D 2001 Variation in the nrDNA ITS of *Pinus* subsection *Cembroides*: implications for molecular systematic studies of pine species complexes. Molecular Phylogenetics and Evolution 21: 449–467. 11741386 10.1006/mpev.2001.1026

[CIT0023] Gernandt DS, Liston A, Piñero D 2003 Phylogenetics of *Pinus* subsections *Cembroides* and *Nelsoniae* inferred from cpDNA sequences. Systematic Botany 28: 657–673.

[CIT0024] Grimm GW, Denk T 2008 ITS evolution in *Platanus* (Platanaceae): homoeologues, pseudogenes and ancient hybridization. Annals of Botany 101: 403–419. 18089582 10.1093/aob/mcm305 PMC2701810

[CIT0025] Gruber B, Unmack PJ, Berry OF, Georges A 2018 dartr: an r package to facilitate analysis of SNP data generated from reduced representation genome sequencing. Molecular Ecology Resources 18: 691–699. 29266847 10.1111/1755-0998.12745

[CIT0026] Gruber B, Unmack P, Berry O, Georges A 2019 Introduction to dartR. https://rdrr.io/cran/dartR/f/inst/doc/IntroTutorial_dartR.pdf. 29 November 2019. 10.1111/1755-0998.12745 29266847

[CIT0027] Holman JE, Hughes JM, Fensham RJ 2003 A morphological cline in Eucalyptus: a genetic perspective. Molecular Ecology 12: 3013–3025. 14629382 10.1046/j.1365-294x.2003.01970.x

[CIT0028] Jaccoud D, Peng K, Feinstein D, Kilian A 2001 Diversity arrays: a solid state technology for sequence information independent genotyping. Nucleic Acids Research 29: E25. 11160945 10.1093/nar/29.4.e25 PMC29632

[CIT0029] Jasińska AK, Wachowiak W, Muchewicz E, Boratyńska K, Montserrat JM, Boratyński A 2010 Cryptic hybrids between *Pinus uncinata* and *P. sylvestris*. Botanical Journal of the Linnean Society 163: 473–485.

[CIT0030] Jombart T 2008 adegenet: a R package for the multivariate analysis of genetic markers. Bioinformatics (Oxford, England) 24: 1403–1405. 10.1093/bioinformatics/btn129 18397895

[CIT0031] Jombart T, Collins C 2015 *A tutorial for discriminant analysis of principal components (DAPC) using adegenet 2.0.* http://adegenet.r-forge.r-project.org/files/tutorial-dapc.pdf. 29 November 2019.

[CIT0032] Jombart T, Devillard S, Balloux F 2010 Discriminant analysis of principal components: a new method for the analysis of genetically structured populations. BMC Genetics 11: 94. doi: 10.1186/1471-2156-11-94. 20950446 10.1186/1471-2156-11-94 PMC2973851

[CIT0033] Kral R 1993 Pinus. In: Flora of North America Editorial Committee, eds. Flora of North America North of Mexico. New York: Oxford University Press, 373–398.

[CIT0034] Lanner RM 1974 *a* A new pine from Baja California and the hybrid origin of *Pinus quadrifolia*. The Southwestern Naturalist 19: 75–95.

[CIT0035] Lanner RM 1974 *b* Natural hybridization between *Pinus edulis* and *Pinus monophyla* in the American southwest. Silvae Genetica 23: 108–116.

[CIT0036] Lanner RM, Phillips AM III 1992 Natural hybridization and introgression of pinyon pines in northwestern Arizona. International Journal of Plant Sciences 153: 250–257.

[CIT0037] Little EL 1968 Two new pinyon varieties from Arizona. Phytologia 17: 329–342.

[CIT0038] Madsen DB, Rhode D 1990 Early Holocene pinyon (*Pinus monophyla*) in the northeastern Great Basin. Quaternary Research 33: 94–101.

[CIT0039] Malusa J 1992 Phylogeny and biogeography of the pinyon pines (*Pinus* subsect. *Cembroides*). Systematic Botany 17: 42–66.

[CIT0040] Melville J, Haines ML, Boysen K, et al 2017 Identifying hybridization and admixture using SNPs: application of the DArTseq platform in phylogeographic research on vertebrates. Royal Society Open Science 4: 161061. 28791133 10.1098/rsos.161061 PMC5541528

[CIT0041] Menon M, Bagley JC, Friedline CJ, et al 2018 The role of hybridization during ecological divergence of southwestern white pine (*Pinus strobiformis*) and limber pine (*P. flexilis*). Molecular Ecology 27: 1245–1260. 29411444 10.1111/mec.14505

[CIT0042] Menon M, Landguth E, Leal-Saenz A, et al 2020 Tracing the footprints of a moving hybrid zone under a demographic history of speciation with gene flow. Evolutionary Applications 13: 195–209. 31892952 10.1111/eva.12795 PMC6935588

[CIT0043] Montes JR, Peláez P, Willyard A, Moreno-Letelier A, Piñero D, Gernandt DS 2019 Phylogenetics of *Pinus* subsection *Cembroides* Engelm.(Pinaceae) inferred from low-copy nuclear gene sequences. Systematic Botany 44: 501–518.

[CIT0044] Muhs DR, Budahn J, Reheis M, Beann J, Skipp G, Fisher E 2007 Airborne dust transport to the eastern Pacific Ocean off southern California: evidence from San Clemente Island. Journal of Geophysical Research: Atmospheres 112: 17.

[CIT0045] Neri J, Wendt T, Palma-Silva C 2018 Natural hybridization and genetic and morphological variation between two epiphytic bromeliads. Aob PLANTS 10: plx061. doi: 10.1093/aobpla/plx061. 29308124 10.1093/aobpla/plx061 PMC5751037

[CIT0046] Pfenninger M, Reinhardt F, Streit B 2002 Evidence for cryptic hybridization between different evolutionary lineages of the invasive clam genus Corbicula (Veneroida, Bivalvia). Journal of Evolutionary Biology 15: 818–829.

[CIT0047] Pritchard JK, Stephens M, Donnelly P 2000 Inference of population structure using multilocus genotype data. Genetics 155: 945–959. 10835412 10.1093/genetics/155.2.945 PMC1461096

[CIT0048] Raj A, Stephens M, Pritchard JK 2014 fastSTRUCTURE: variational inference of population structure in large SNP data sets. Genetics 197: 573–589. 24700103 10.1534/genetics.114.164350 PMC4063916

[CIT0049] Rieseberg LH, Ellstrand NC, Arnold M 1993 What can molecular and morphological markers tell us about plant hybridization? Critical Reviews in Plant Sciences 12: 213–241.

[CIT0050] Rieseberg LH, Willis JH 2007 Plant speciation. Science 317: 910–914. 17702935 10.1126/science.1137729 PMC2442920

[CIT0051] Rutherford S, Rossetto M, Bragg JG, et al 2018 Speciation in the presence of gene flow: population genomics of closely related and diverging Eucalyptus species. Heredity 121: 126–141. 29632325 10.1038/s41437-018-0073-2 PMC6039520

[CIT0052] Snajberk K, Zavarin E, Debry R 1982 Terpenoid and morphological variability of *Pinus quadrifolia* and the natural hybridization with *Pinus monophyla* in the San Jacinto Mountains in California. Biochemical Systematics and Ecology 10: 121–132.

[CIT0053] Soltis PS, Soltis DE 2009 The role of hybridization in plant speciation. Annual Review of Plant Biology 60: 561–588. 10.1146/annurev.arplant.043008.092039 19575590

[CIT0054] Twyford AD, Ennos RA 2012 Next-generation hybridization and introgression. Heredity 108: 179–189. 21897439 10.1038/hdy.2011.68 PMC3282392

[CIT0055] USGS 1999 *Digital representation of ‘Atlas of United States Trees’.* https://archive.usgs.gov/archive/sites/www.usgs.gov/science/cite-view.php-cite=22.html. 29 November 2019.

[CIT0056] Von Mark VC, Kilian A, Dierig DA 2013 Development of DArT marker platforms and genetic diversity assessment of the U.S. collection of the new oilseed crop lesquerella and related species. PLoS One 8: e64062. doi: 10.1371/journal.pone.0064062. 23724020 10.1371/journal.pone.0064062 PMC3665832

[CIT0057] Wei L, Li YF, Zhang H, Liao WJ 2015 Variation in morphological traits in a recent hybrid zone between closely related *Quercus liaotungensis* and *Q. mongolica* (Fagaceae). Journal of Plant Ecology 8: 224–229.

[CIT0058] Wells PV 1983 Paleobiogeography of montane islands in the Great Basin since the last glaciopluvial. Ecological Monographs 53: 341–382.

[CIT0059] Wenzl P, Carling J, Kudrna D, et al 2004 Diversity Arrays Technology (DArT) for whole-genome profiling of barley. Proceedings of the National Academy of Sciences, USA 101: 9915–9920. 10.1073/pnas.0401076101 PMC470773 15192146

[CIT0060] Williams CG 2010 Long-distance pine pollen still germinates after meso-scale dispersal. American Journal of Botany 97: 846–855. 21622450 10.3732/ajb.0900255

[CIT0061] Willyard A, Cronn R, Liston A 2009 Reticulate evolution and incomplete lineage sorting among the ponderosa pines. Molecular Phylogenetics and Evolution 52: 498–511. 19249377 10.1016/j.ympev.2009.02.011

[CIT0062] Wright JW 1952 Pollen dispersion of some forest trees. Northeastern Forest Experiment Station Papers 46: 1–42.

[CIT0063] Xiao LQ, Möller M, Zhu H 2010 High nrDNA ITS polymorphism in the ancient extant seed plant Cycas: incomplete concerted evolution and the origin of pseudogenes. Molecular Phylogenetics and Evolution 55: 168–177. 19945537 10.1016/j.ympev.2009.11.020

[CIT0064] Zavarin E, Snajberk K, Debry R 1980 Terpenoid and morphological variability of *Pinus quadrifolia* and its natural hybridization with *Pinus monophyla* in northern Baja California and adjoining United States. Biochemical Systematics and Ecology 8: 225–235.

[CIT0065] Zhao W, Meng J, Wang B, et al 2014 Weak crossability barrier but strong juvenile selection supports ecological speciation of the hybrid pine *Pinus densata* on the Tibetan plateau. Evolution 68: 3120–3133. 25065387 10.1111/evo.12496 PMC4278550

