# Supplementary figures and images for: Patterns of hybridization and cryptic introgression among one- and four-needled pinyon pines
Source: Ann Bot. 2020 Mar 28;126(3):401–11. doi: 10.1093/aob/mcaa045 (PMC7424738; doi:10.1093/aob/mcaa045)

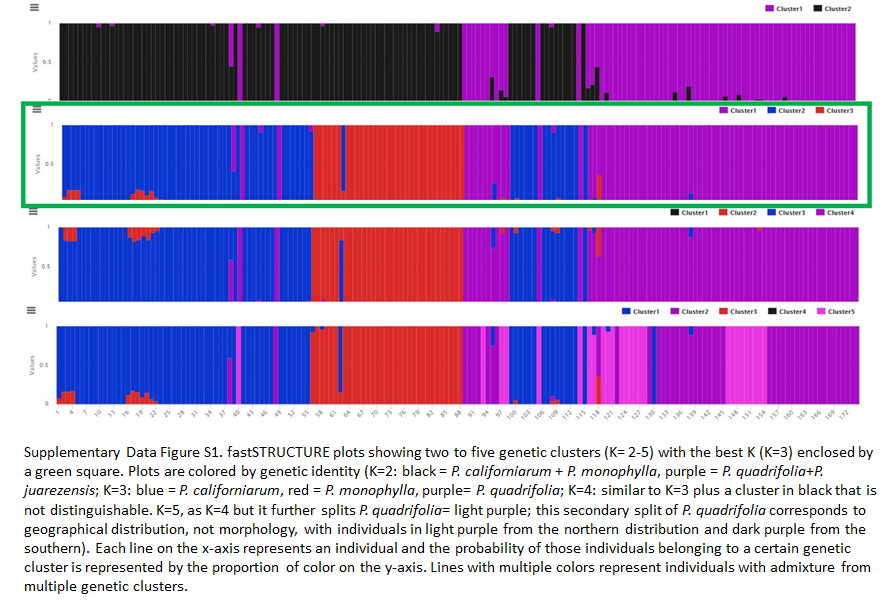

Supplement: mcaa045_suppl_Supplementary_Figure_1 [file mcaa045_suppl_supplementary_figure_1.png]

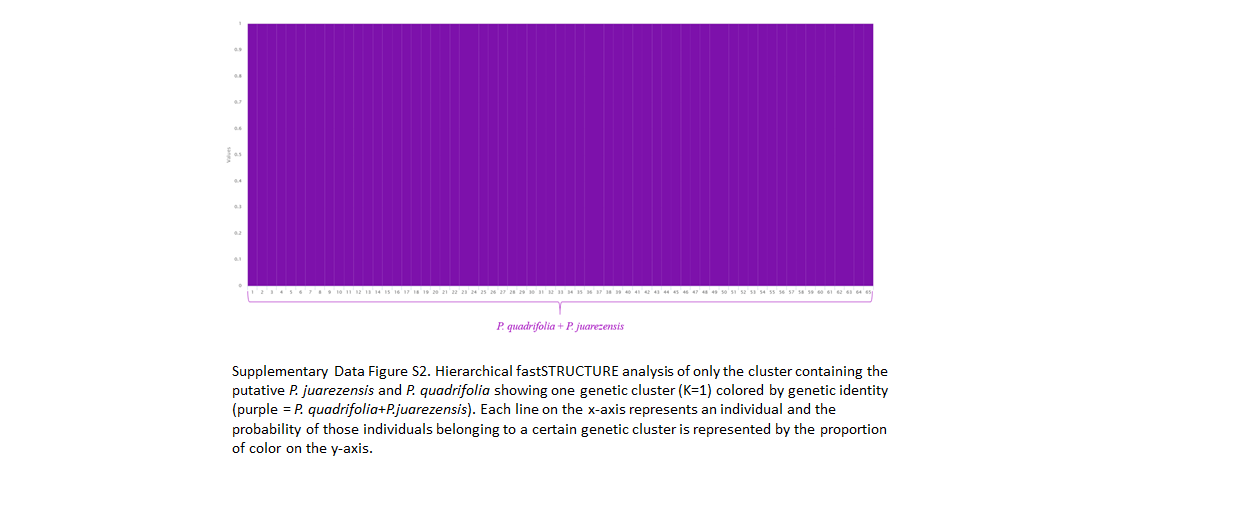

Supplement: mcaa045_suppl_Supplementary_Figure_2 [file mcaa045_suppl_supplementary_figure_2.png]

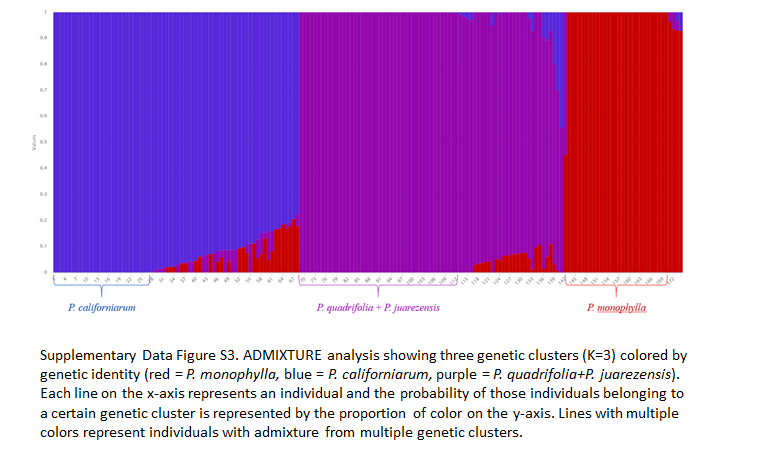

Supplement: mcaa045_suppl_Supplementary_Figure_3 [file mcaa045_suppl_supplementary_figure_3.png]
